# Research on the influencing factors of generative artificial intelligence usage intent in post-secondary education: an empirical analysis based on the AIDUA extended model

**DOI:** 10.3389/fpsyg.2025.1644209

**Published:** 2025-09-17

**Authors:** Xueyan Bai, Lin Yang

**Affiliations:** School of Journalism and New Media, Xi'an Jiaotong University, Xi'an, China

**Keywords:** post-secondary education, generative artificial intelligence, technology acceptance, multi-group analysis, AIDUA model

## Abstract

**Objective:**

Generative Artificial Intelligence (AIGC) presents a profound dialectic in higher education: its transformative potential is challenged by deep-seated psychological and ethical barriers. Traditional adoption models fail to capture this complexity. To bridge this gap, this study develops and tests an integrated cognitive-behavioral framework. We posit that AIGC acceptance is a three-stage cognitive appraisal process. By embedding an extended AIDUA model—a framework specifically tailored to the unique challenges of AI adoption—within Cognitive Appraisal Theory, we investigate how novel antecedent dimensions (Socio-Ethical: ethical risk, explainability; Techno-Performance: generation quality, context-awareness) and classical factors (social influence, hedonic motivation, anthropomorphism) shape core technological beliefs (Performance & Effort Expectancy), which in turn mediate the path to acceptance intention via emotion. Furthermore, the moderating roles of gender, academic background, ethnicity, and political affiliation are systematically examined to test the model’s boundary conditions.

**Methods:**

The model was empirically validated using Structural Equation Modeling and multi-group analysis on survey data from 462 university students across 15 diverse institutions in China.

**Results:**

The findings reveal that the cognitive appraisal of AIGC is primarily driven by its perceived capabilities and safety. Techno-Performance (generation quality, *β* = 0.53) and Socio-Ethical (explainability, *β* = 0.41; ethical risk, *β* = −0.25) dimensions were the most powerful predictors of Performance Expectancy. These intrinsic appraisals significantly outweighed the influence of external social cues. Notably, ethical risk perception operated as a dual-threat, not only lowering performance expectations but also significantly amplifying the perceived cognitive burden (Effort Expectancy, *β* = 0.33). Multi-group analyses confirmed that these appraisal pathways are systematically moderated by individual and cultural background variables, highlighting significant heterogeneity in user responses.

**Discussion:**

This study makes a critical theoretical contribution by demonstrating how core technological expectancies are formed through a multi-stage appraisal of utility, ethics, and experience, moving beyond mere identification of influential factors. The findings dismantle the myth of a universal “student user,” revealing that AIGC adoption is a culturally and contextually embedded process. Practically, the results provide an evidence-based roadmap for university policymakers and AIGC developers, emphasizing that fostering trust and adoption requires a dual focus: maximizing technological prowess while actively mitigating perceived ethical and cognitive costs through enhanced transparency and user-centric design.

## 1 Introduction

Generative Artificial Intelligence (AIGC) is a disruptive technological paradigm profoundly reshaping the landscape of modern education (Chen et al., 2024). Its core systemic attribute—operating as a probabilistic, rather than deterministic, information generator—creates a fundamental distinction from all prior educational technologies. This distinction gives rise to an inherent contradiction in its application: while AIGC can catalyze personalized learning (Huangfu et al., 2025), its intrinsic opacity and unpredictability concurrently fuel deep-seated concerns among scholars and practitioners regarding data privacy (Wang et al., 2024), algorithmic bias (Fang et al., 2024), and informational reliability (Cui and Zhang, 2025).

Consequently, traditional technology acceptance models, such as the Technology Acceptance Model (TAM), which were designed to evaluate deterministic tools, encounter a theoretical bottleneck (Wong et al., 2024). The core logic of these models—anchored in perceived usefulness and perceived ease of use—fails to adequately capture the cognitive trade-off individuals perform when confronting AIGC, a complex deliberation between the opportunities of technological empowerment and the specter of potential ethical risks. This trade-off process, rather than a simple utilitarian calculus, is the crux of understanding AIGC adoption behavior. Therefore, a primary challenge in the current research landscape is the lack of a theoretical framework capable of effectively elucidating the cognitive mechanisms that underpin this trade-off.

However, as a high-level meta-theory, Cognitive Appraisal Theory (CAT) defines the process of appraisal but does not furnish the specific content variables applicable to a given technological context. To this end, a behavioral model is required to operationalize its theoretical constructs. This study selects the Acceptance of Artificial Intelligence and Data Analytics (AIDUA) model (Gursoy et al., 2019) as its theoretical foundation precisely because it emerged from the previous wave of AI, characterized by big data and machine learning, and was designed to overcome the limitations of TAM in explaining more complex technologies (Lin et al., 2020). To achieve this, AIDUA incorporates not only the core utilitarian predictors of Performance Expectancy and Effort Expectancy but also integrates a suite of non-utilitarian antecedents aimed at capturing the richness of human motivation, such as Social Influence (Hu et al., 2019), Hedonic Motivation (Tamilmani et al., 2019), and Anthropomorphism (Ding et al., 2022).

Yet, we must critically recognize that the “artificial intelligence” targeted by the AIDUA model at its inception is fundamentally different from the generative paradigm of AIGC we face today. AIDUA was primarily developed for analytical AI (e.g., big data analytics, business intelligence), whose function is to process and interpret existing information, rendering its risks relatively manageable. For this reason, it carries the theoretical DNA of its parent theory, the Unified Theory of Acceptance and Use of Technology (UTAUT). UTAUT was originally designed to predict user adoption of traditional information systems with deterministic functions and controllable risks, such as enterprise resource planning systems (Williams et al., 2015). Within such a “benign tool” evaluation framework, the technology’s performance is an implicitly stable premise, and ethical risks are not a central consideration for the user.

Therefore, AIDUA’s existing set of antecedents (Social Influence, Hedonic Motivation, Anthropomorphism, etc.) is, in essence, a variable set designed to explain “how to better accept an analytical tool with clear functional boundaries.” However, when the object of evaluation shifts from analytical AI to the AIGC we confront today—a probabilistic, high-risk “creative agent” capable of generating entirely new content—the explanatory power of this variable set reveals its fundamental limitations. According to the tenets of CAT, the core of Primary Appraisal lies in the individual’s trade-off between the Opportunity (Challenge) and Threat dimensions of a stimulus (in this case, AIGC). AIDUA’s current variables are clearly insufficient for this task, leaving theoretical blind spots in two critical areas: (1) a lack of direct assessment of “technical efficacy,” which is the core judgment of the opportunity dimension (i.e., AIGC’s performance) (Yao et al., 2025); and (2) a lack of systematic consideration of “emergent risks,” the key evaluation of the threat dimension (i.e., potential ethical and algorithmic issues) (Xie, 2025). It can therefore be asserted that the AIDUA model’s existing set of antecedents is incomplete. The theoretical necessity of the two new dimensions proposed in this study, “Techno-Performance” and “Socio-Ethical,” lies precisely here: they are not redundant with or replacements for the existing variables, but rather parallel and essential supplements intended to fill these identified theoretical voids.

Finally, CAT repeatedly emphasizes that any cognitive appraisal does not occur in a vacuum but is systematically moderated by the personal experiences and sociocultural contexts in which an individual is embedded (Kuh and Hu, 2001). Consequently, examining moderating effects is not an optional add-on for this study but a requisite for ensuring theoretical integrity and exploring the model’s boundary conditions. To this end, this study employs multi-group analysis to test the stability of our proposed cognitive-behavioral path model across different sub-populations. In selecting moderating variables, we follow a logical hierarchy from general individual traits to organizationally and culturally specific ones. First, at the level of general individual characteristics, we select the classic variables of Gender and Academic Background (Qazi et al., 2022). Further, we investigate variables that reflect an individual’s deep-seated identity within China’s specific social fabric: namely, Ethnicity and Political Affiliation.

Regarding Ethnicity (Han vs. Ethnic Minorities): Within China’s multi-ethnic state framework, this variable is often correlated with systemic differences in upbringing, accessibility of educational resources, and channels of information exposure (Jin and Liang, 2015), offering a unique window through which to observe the impact of cultural capital.

Regarding Political Affiliation (Communist Party of China Member vs. Non-Member): In the Chinese context, this variable is more than a political identity; it reflects an individual’s degree of alignment with mainstream institutional values and their level of integration into established information networks (Huang et al., 2025).

We hypothesize that the profound socioeconomic and cultural differences represented by these variables will systematically moderate the opportunity-threat trade-off process that users undertake when facing AIGC.

In summary, this study aims to systematically unveil the complex decision-making mechanisms underlying user adoption of AIGC in educational contexts by developing and validating an extended AIDUA model integrated with CAT. Through the construction of this theoretical model and the examination of its multi-group moderating effects, this research seeks not only to reveal a main-effects model that holds “on average” but also to paint a fine-grained panoramic picture, co-regulated by individual identity and institutional affiliation. In doing so, it aims to provide profound theoretical insights into the social acceptance process of this transformative technology. The remainder of this paper is structured as follows: First, we will conduct a literature review and elaborate on the theoretical foundations of our proposed model and its research hypotheses. Next, we will introduce the research methodology. Subsequently, the data analysis results will be presented. Finally, the discussion section will offer an in-depth interpretation of the study’s theoretical contributions and practical implications.

## 2 Theoretical basis and research hypotheses

### 2.1 Theoretical framework: a hierarchical model integrating cognitive appraisal and an extended AIDUA framework

#### 2.1.1 The foundational content framework and its procedural limitation: the AIDUA model

To investigate the acceptance of AIGC, this study adopts AIDUA model as its foundational content framework. Developed specifically to address the unique characteristics of AI-driven systems, the AIDUA model provides the established core constructs for our study. Specifically, from this model, we derive the central belief variables of performance expectancy and effort expectancy; the key antecedent factors of social influence, hedonic motivation, and anthropomorphism; and the critical outcome of emotion which precedes final acceptance. This set of variables offers a comprehensive initial blueprint for evaluating multifaceted, interactive technologies like AIGC, serving as the backbone of our research framework (Begum et al., 2025).

However, while the AIDUA model posits a valuable macro-level sequence (i.e., antecedents influencing core beliefs, which in turn shape intentions), it exhibits a mechanistic limitation. The model powerfully demonstrates that factors like social influence affect performance expectancy, but it does not fully elaborate on the underlying psychological mechanism of how this influence is cognitively processed. The arrow from an antecedent to a core belief remains a “black box.” It provides a structural pathway but lacks a deep explanatory theory for the cognitive transformations occurring along that pathway. Given that AIGC adoption is a complex process of appraisal and reaction, merely identifying influential pathways is insufficient; a more granular, theory-driven explanation of the user’s cognitive journey is required.

#### 2.1.2 The overarching process framework: the integration of cognitive appraisal theory

To address this mechanistic limitation, this study integrates CAT, not to replace the structure of AIDUA, but to provide it with a micro-level explanatory mechanism. The core contribution of CAT lies in its ability to “unpack” the black-boxed relationships within the AIDUA framework. The theory’s central tenet—the sequential interplay between primary appraisal (evaluating what is at stake) and secondary appraisal (evaluating coping potential)—offers a detailed account of how external cues, such as a peer’s recommendation, are psychologically translated into a core belief, such as “this AIGC is useful” (So et al., 2016). This provides the explanatory depth lacking in the original model.

Furthermore, CAT affords excellent theoretical extensibility for model expansion. Its core concept of “appraisal” not only licenses the introduction of new variables but also logically necessitates the identification of informational inputs that are most decisive to user evaluations within a specific technological context. As articulated in the introduction, when the object of evaluation shifts from a “deterministic analytical tool” to a “creative agent” capable of generating novel content, the focal point of user appraisal undergoes a fundamental shift. According to the theoretical precepts of CAT, a user’s primary appraisal will inevitably revolve around the fundamental dimensions of “opportunity/challenge” and “threat” posed by the AIGC. The established antecedents in AIDUA leave two theoretical gaps in this regard, which precisely establishes the theoretical necessity for introducing new dimensions in this study:

The Core of the Opportunity Dimension—The Technical Performance Dimension: The “core product” of an AIGC is the content it generates. Consequently, a user’s primary appraisal of its utility will invariably focus on the quality and relevance of its outputs. This constitutes the most direct and objective evidence for a benefit appraisal. We therefore introduce the Technical Performance Dimension, operationalized through Generation Quality (Zhou et al., 2025) and Context-awareness (Wei, 2024), to capture the user’s assessment of the core “opportunity” presented by the AIGC.

The Core of the Threat Dimension—The Socio-Ethical Dimension: When a technology begins to “create” autonomously, it evolves from a passive tool into an active “social actor,” which invariably triggers a user’s threat appraisal concerning its potential societal consequences. We therefore introduce the Socio-Ethical Dimension, operationalized through Perceived Ethical Risk (Zhou et al., 2024) and Algorithmic Explainability (Ződi, 2022), to capture the user’s cognitive and coping evaluations when faced with this new category of “threat.”

Therefore, the integration of these two dimensions is not an arbitrary addition. Instead, it is a theoretically-driven and necessary response to the fundamental change in the nature of AIGC technology, as mandated by CAT. They serve as critical supplements, parallel to the classic AIDUA antecedents, to collectively form a more complete set of informational inputs essential for the user’s cognitive appraisal process.

#### 2.1.3 The final integrated model: extending the framework for the AIGC context

By integrating the AIDUA-derived content variables with the CAT-justified new dimensions, we construct the final, extended model for this study. To articulate the logic of this model with maximum clarity, we conceptualize it as a conceptually hierarchical framework.

This structure is not arbitrary; it is logically derived from the foundational causal chain inherent in both TAMs and cognitive psychology, which progresses from external stimuli to cognitive processing, and finally to belief formation and behavioral response. We deconstruct this progression into four distinct analytical layers. It is crucial to note that while these layers are presented sequentially for theoretical explanation, the underlying cognitive activities—particularly between Layer 1 and 2—are often instantaneous and iterative in reality.

Layer 1: Antecedent Informational Cues (The Stimuli). This layer comprises all external and internal factors that provide salient information to the user. In our model, this includes social influence, hedonic motivation, anthropomorphism, and the AIGC-specific technical performance and socio-ethical dimensions. This layer answers the question: What is being appraised?
Layer 2: Cognitive Appraisal Mechanism (The Processing). This is the core explanatory layer, governed by CAT. It is not represented by variables, but by the causal paths from Layer 1 to Layer 3, detailing how the informational cues are processed through primary and secondary appraisal.
Layer 3: Core Belief Formation (The Immediate Cognitive Response). This layer represents the direct outputs of the appraisal process—the user’s refined judgments. In our model, these are performance expectancy and effort expectancy.
Layer 4: Affective and Behavioral Outcomes (The Final Response). This final layer includes the subsequent emotional reaction (emotion) and the ultimate behavioral disposition (acceptance intention).

This layered architecture demonstrates both synergy—the AIDUA model provides the core content for appraisal, while CAT provides the process of appraisal—and a clear hierarchy. It moves beyond a simple associative model by providing a structured, in-depth explanation of the psychological pathway from initial cue evaluation to final behavioral intent. This explicit mapping provides a solid foundation for the subsequent development of our research hypotheses.

### 2.2 Hypothesis development: mechanistic deduction within the CAT framework

This section systematically develops the research hypotheses by strictly adhering to the four-layer hierarchical framework established in Section 2.1. The deduction process explicitly demonstrates how the Cognitive Appraisal Mechanism (Layer 2) processes the Antecedent Informational Cues (Layer 1) to produce the Core Beliefs (Layer 3). Finally, it connects these beliefs to the Affective and Behavioral Outcomes (Layer 4). The hypotheses are organized into logical clusters that mirror this causal path, ensuring that each proposed relationship is a direct output of the CAT-governed theoretical engine.

#### 2.2.1 Cluster 1: the influence of antecedent cues on core beliefs

This first and most substantial cluster of hypotheses details the core cognitive appraisal process. For each antecedent variable (a Layer 1 cue), we will explain how it is processed through primary appraisal (evaluating stakes) and secondary appraisal (evaluating coping potential)—the core Layer 2 mechanism—to shape a user’s foundational beliefs about AIGC’s utility (Performance Expectancy) and its usability (Effort Expectancy) (Layer 3).

##### 2.2.1.1 The classic AIDUA antecedents as appraisal inputs

We begin with the established factors derived from the AIDUA model, reinterpreting their influence through the lens of cognitive appraisal.

###### 2.2.1.1.1 Social influence

Social influence provides critical external social cues that are processed by the user (Kulviwat et al., 2009). In primary appraisal, positive signals from peers or experts are evaluated as evidence of situational benefit, framing the use of AIGC as a valuable and socially desirable action. This appraisal enhances its perceived utility and leads to higher performance expectancy (Figueroa-Armijos et al., 2023). Concurrently, in secondary appraisal, the successful experiences of others (vicarious learning) are evaluated as a signal of high coping potential. This evaluation leads to the belief that one can also master the technology with reasonable effort, thus resulting in a lower effort expectancy (Chua et al., 2018).

*H1*: Social influence positively influences performance expectancy.

*H2*: Social influence negatively influences effort expectancy.

###### 2.2.1.1.2 Hedonic motivation

The intrinsic fun or pleasure derived from using a technology acts as a powerful affective input (Al-Azawei and Alowayr, 2020). During primary appraisal, the anticipation of enjoyment is evaluated as a significant benefit in itself, an appraisal that increases the technology’s overall performance expectancy beyond mere utilitarian goals (Sitar-Tăut, 2021). During secondary appraisal, this intrinsic appeal is evaluated as a factor that transforms potential cognitive “costs” into an enjoyable challenge. This evaluation enhances perceived coping resources and reduces perceived difficulty, leading to a lower effort expectancy (Ramírez-Correa et al., 2019).

*H3*: Hedonic motivation positively influences performance expectancy.

*H4*: Hedonic motivation negatively influences effort expectancy.

###### 2.2.1.1.3 Anthropomorphism

Attributing human-like characteristics to AIGC reframes the technology from a “tool” to a “partner,” which fundamentally alters its appraisal (Jia et al., 2021). In primary appraisal, a “partner” is evaluated as being more capable and agentic than a simple tool, an appraisal that enhances the perception of its potential benefits and leads to higher performance expectancy (Tian and Wang, 2022). In secondary appraisal, interaction with a human-like agent is evaluated as more natural and intuitive. This evaluation of lower cognitive cost reduces the anticipated difficulty of use, resulting in a lower effort expectancy (Moriuchi, 2021).

*H5*: Anthropomorphism positively influences performance expectancy.

*H6*: Anthropomorphism negatively influences effort expectancy.

##### 2.2.1.2 The AIGC-specific dimensions as appraisal inputs

Next, we integrate the two new dimensions critical to the AIGC context, detailing how their unique informational cues are processed by the appraisal mechanism.

###### 2.2.1.2.1 The socio-ethical dimension

This dimension captures appraisals of threat and uncertainty. Perceived ethical risk (Guan et al., 2022) acts as a primary input for appraising potential “threats,” while algorithmic explainability (Shin, 2021) is key to appraising and reducing “uncertainty.” In primary appraisal, high ethical risk is evaluated as a significant threat that directly undermines any potential benefits, thus negatively impacting performance expectancy. In parallel, high explainability is evaluated as a mitigator of uncertainty, which builds trust and, in turn, enhances the perception of the system’s value (Gînguță et al., 2023). In secondary appraisal, high ethical risk is appraised as increasing the cognitive burden of coping (e.g., requiring constant vigilance), thus leading to a higher effort expectancy. Conversely, an explainable and predictable system is evaluated as being more controllable, an appraisal that significantly reduces the mental effort needed to use it effectively and results in a lower effort expectancy (Rahi et al., 2019).

*H7*: Perceived ethical risk negatively influences performance expectancy.

*H8*: Perceived ethical risk positively influences effort expectancy.

*H9*: Algorithmic explainability positively influences performance expectancy.

*H10*: Algorithmic explainability negatively influences effort expectancy.

###### 2.2.1.2.2 The technical performance dimension

This dimension, encompassing generation quality (Zhang et al., 2023) and context-awareness (Shi et al., 2025), provides the most direct, objective evidence for appraisal. In primary appraisal, high technical performance is evaluated as clear proof of the system’s utility, strongly supporting a positive benefit judgment and leading to higher performance expectancy (Steiss et al., 2024). In secondary appraisal, a system that produces accurate and context-aware results is appraised as reducing the user’s need for constant corrections and mental adjustments. This evaluation drastically lowers the perceived cognitive costs and thus results in a lower effort expectancy (Singh et al., 2025).

*H11*: Generation quality positively influences performance expectancy.

*H12*: Generation quality negatively influences effort expectancy.

*H13*: Context-awareness positively influences performance expectancy.

*H14*: Context-awareness negatively influences effort expectancy.

#### 2.2.2 Cluster 2: the path from core beliefs (layer 3) to final outcomes (layer 4)

This final cluster maps the progression from cognitive judgment to emotional and behavioral responses, completing the causal chain of the model. According to CAT, cognitive judgments logically precede emotional reactions (Schwoerer et al., 2005). A positive judgment of utility (performance expectancy) and ease (low effort expectancy) should elicit positive emotions like satisfaction and confidence (Hong et al., 2023). This positive affective state, in turn, facilitates an approach-oriented behavioral response, manifesting as the ultimate intention to accept and use the technology (Jeon et al., 2020).

*H15*: Performance expectancy positively influences emotion.

*H16*: Effort expectancy negatively influences emotion.

*H17*: Emotion positively influences acceptance intention.

*H18*: Performance expectancy positively influences acceptance intention.

*H19*: Effort expectancy positively influences acceptance intention.

### 2.3 The moderating role of individual differences: justification for multi-group analysis

While the proposed integrated framework delineates the core psychological mechanisms of AIGC acceptance, these processes do not occur in a social vacuum. To test the boundary conditions of our model and deepen its explanatory power in the unique Chinese context, we propose a multi-group analysis. The choice of moderators—gender, academic background, ethnicity, and political affiliation—is deliberate. Each represents a key social identity that, within China’s specific socio-cultural structure, systematically shapes an individual’s cognitive framework and value hierarchy, thereby directly addressing the reviewer’s concern about their contextual relevance (Venkatesh et al., 2012).

#### 2.3.1 Gender

Gender differences in technology perception are well-documented globally, and this holds true in China. Traditionally, studies have reported that men tend to be more influenced by instrumental factors like perceived usefulness (performance expectancy), while women may place greater weight on ease of use (effort expectancy) and social influence (Venkatesh et al., 2000). More recently, research in the AI context has highlighted that women often express higher levels of ethical concern and perceive greater risks associated with algorithmic decision-making (Adewale, 2025). These differences provide a foundational, universally accepted baseline for moderation analysis.

#### 2.3.2 Academic background

In the context of China’s highly structured education system, the distinction between STEM and Humanities/Social Sciences is not merely a difference in knowledge, but a cultivation of divergent cognitive paradigms. Students from STEM (Science, Technology, Engineering, and Mathematics) fields are trained in a utility-focused, problem-solving framework, likely amplifying the importance of performance-related appraisals (e.g., generation quality) (Caldeira et al., 2021). Conversely, students from the humanities and social sciences are explicitly trained to be more critical of the socio-ethical dimensions of any phenomenon, making their acceptance far more sensitive to perceived ethical risks and algorithmic explainability (Ilomäki and Lakkala, 2018). This makes academic background a meaningful and powerful moderator in this context.

#### 2.3.3 Ethnicity

In China, a multi-ethnic nation, ethnicity is a crucial dimension of social identity tied to distinct cultural heritages and collective narratives. For the Han majority, who are deeply integrated into the mainstream technological and commercial milieu, the appraisal of a new technology like AIGC is often focused on individual utility. However, for many ethnic minority groups, a primary concern is the preservation of their unique cultural identity and the accurate representation of their collective image. Given that AIGC can perpetuate or even amplify cultural stereotypes, minority users are rationally more sensitive to this collective cultural risk (Noble, 2018). This heightened sensitivity means that the Socio-Ethical Dimension (e.g., algorithmic fairness, cultural respect) is not an abstract concern but a direct factor in their appraisal calculus, making ethnicity a highly relevant moderator for this topic in China (Rainie and Anderson, 2017).

#### 2.3.4 Political affiliation

**Figure 1:**
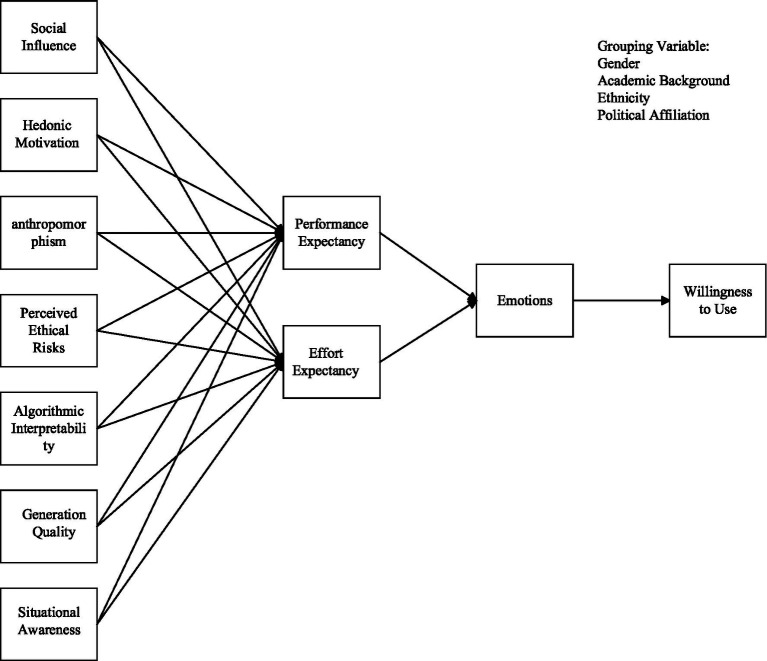
Theoretical model.

In China, membership in the Communist Party of China (CPC) is a unique social identity that fundamentally shapes an individual’s cognitive framework beyond a mere political label. It fosters a heightened sense of social responsibility and alignment with national strategic objectives (Bian et al., 2001). Consequently, we propose that CPC and non-CPC members employ divergent cognitive calculi when appraising AIGC. Non-members’ evaluations are likely anchored in a primary calculus of personal utility. Conversely, we theorize that CPC members adopt a dual-calculus perspective, integrating personal utility with a secondary, socio-political assessment. For this group, higher-order considerations, particularly those in the Socio-Ethical Dimension (e.g., content alignment with societal values, potential for misuse, contribution to national innovation), are weighted more heavily, reflecting an appraisal process intrinsically linked to collective interests (Brødsgaard, 2012). This makes political affiliation a theoretically innovative and contextually vital moderator (see Figure 1).

## 3 Research design and methodology

This study aims to develop and validate an integrated theoretical model to investigate the key antecedents, cognitive appraisal processes, and affective mechanisms influencing Chinese university students’ intention to accept and use AIGC. To achieve this, the study employs a quantitative research approach, utilizing a questionnaire survey to collect data, which is then empirically analyzed using Structural Equation Modeling (SEM) and Multi-Group Analysis. A cross-sectional design was adopted to capture students’ perceptions and attitudes regarding AIGC at a specific point in time.

### 3.1 Participants and sampling procedure

The target population for this research was university students in China. To ensure the breadth and representativeness of the sample, a multi-stage stratified random sampling strategy was employed. First, we operationalized the stratification by categorizing universities into nine strata based on a 3 (Region: Eastern, Central, Western) × 3 (Type: Comprehensive, Science & Engineering, Normal) matrix. This stratification was theoretically motivated by documented regional economic disparities and distinct disciplinary cultures across Chinese higher education (Yang et al., 2014), factors that could substantively influence technology adoption patterns. From each stratum, we then randomly selected one to two universities, resulting in our final roster of 15 institutions. Within each selected university, the questionnaire link was then distributed through academic affairs offices or student advisors to a random selection of students across various disciplines, ensuring heterogeneity in academic backgrounds.

The survey was administered via “Wenjuanxing,” a professional online questionnaire platform. Between June and October 2024, a total of 480 questionnaires were distributed. Prior to participation, all potential respondents were informed of the research objectives, the voluntary nature of their participation, and data confidentiality measures, and were required to provide online informed consent. To guarantee data quality, stringent screening criteria were established: (1) participants had to be full-time enrolled students aged 18 or older; (2) completion time was too short (e.g., less than 180 s); and (3) there were evident patterns of regular or repetitive responses. After data cleaning, 18 invalid questionnaires were excluded, resulting in a final sample of 462 valid responses. This constitutes a high effective response rate of 96.3%. The final sample was reasonably balanced across the 15 participating institutions, preventing any single institution from dominating the dataset.

The demographic characteristics of the final sample are detailed in Table 1. The sample demonstrates good diversity in terms of gender, grade level, and academic background, providing a solid foundation for the analysis.

**Table 1:** Demographic characteristics of the sample.

Characteristics	Category	Frequency	Percentage
Gender	Male	226	48.9
	Female	236	51.1
Academic background	Humanities and social sciences	211	45.7
	Science and engineering	251	54.3
Ethnic group	Han	418	90.5
	Minorities	44	9.5
Political affiliation	Party member	267	57.8
	Non-party member	195	42.2
Total	462	462	100.0

### 3.2 Instrument development and measures

The survey instrument consisted of two sections: demographic information and the measurement scales for the core constructs. All core constructs were measured using items adapted from established and widely validated English-language scales to ensure their theoretical grounding and content validity.

The scale adaptation and translation process strictly followed the cross-cultural research paradigm. First, the original English items were independently translated into Chinese by two bilingual doctoral students (one majoring in educational technology, the other in psychometrics). The research team then convened to reconcile any discrepancies. Subsequently, a back-translation was conducted by a linguistic expert unfamiliar with the original scales to verify translational equivalence.

Crucially, prior to the main survey, a pilot study was conducted with 30 university students who were not part of the final sample to assess the clarity of the items and the initial reliability of the constructs. Feedback led to minor wording adjustments, and the initial Cronbach’s alpha values for all constructs were above the recommended 0.70 threshold, providing confidence for the large-scale survey.

All measurement items were rated on a five-point Likert scale, ranging from “1 = Strongly Disagree” to “5 = Strongly Agree.” Recognizing that constructs such as Perceived Ethical Risks may be susceptible to social desirability bias, several procedural remedies were embedded in the data collection process, as detailed in Section 3.4. The specific sources for the scales and a full list of measurement items are provided in Table 2.

**Table 2:** Variables and problems.

Variable	Item	References
Social influence	1. My classmates/friends have a positive attitude toward AIGC technology.	Abdaljaleel et al. (2024)
	2. I feel supported by my peers, which enhances my expectations of AIGC technology.	
	3. In my social circle, the use of AIGC technology is considered beneficial.	
Hedonic motivation	1. I believe using AIGC technology can provide an enjoyable learning experience.	Abdaljaleel et al. (2024)
	2. I look forward to using AIGC technology to gain enjoyment in my learning.	
	3. I have a high level of interest in using such technology.	
Anthropomorphism	1. I believe that if AIGC technology is designed to be more human-like, it would enhance my learning outcomes.	Zhang and Rau (2023)
	2. AIGC technology with human-like characteristics raises my expectations of its performance.	
	3. I am more optimistic about the results of using a personified AIGC system.	
Perceived ethical risks	1. I am concerned that using AIGC technology may raise ethical issues.	Stahl and Eke (2024)
	2. My perception of the ethical risks of AIGC technology affects my expectations of its effectiveness.	
	3. If I have concerns about the ethical risks of AIGC technology, I will lower my expectations of it.	
Algorithmic interpretability	1. If I can understand how AIGC technology works, I would trust its output more.	Chen (2024)
	2. Clear algorithmic interpretability raises my expectations for the effectiveness of AIGC technology.	
	3. I hope AIGC technology will provide transparent operation and result explanations.	
Generation quality	1. I expect AIGC technology to deliver high-quality results.	Sančanin and Penjišević (2022)
	2. I believe the quality of generated content directly impacts my learning outcomes.	
	3. High-quality outputs will enhance my expectations for using AIGC technology.	
Context-awareness	1. AIGC technology can provide targeted assistance based on my learning context.	Augusto (2022)
	2. I expect AIGC technology to understand and adapt to my learning needs.	
	3. AIGC technology with strong Context-awareness makes me more confident in its effectiveness.	
Performance expectancy	1. I believe AIGC technology can improve my learning efficiency.	Elliot et al. (2021)
	2. I am confident in the effectiveness of AIGC technology.	
	3. I expect that using AIGC technology will lead to significant learning outcomes.	
Effort expectancy	1. I feel that using AIGC technology will be an easy and pleasant experience.	Cao and Niu (2019)
	2. The experience of using AIGC technology makes me willing to put in more effort.	
	3. I am willing to invest time and effort to learn AIGC technology.	
Emotions	1. I feel excited and positive when using AIGC technology.	Venkatesh et al. (2003)
	2. The emotions I experience while using AIGC technology are pleasant.	
	3. I am satisfied with my experience using AIGC technology.	
Willingness to use	1. I am willing to try using AIGC technology for learning.	Gursoy et al. (2019)
	2. I hope to continue using AIGC technology in the future.	
	3. I would recommend AIGC technology to my peers.	

### 3.3 Data analysis strategy

Data analysis was performed using SPSS 27.0 and AMOS 24.0 software. The analysis proceeded in the following stages:

#### 3.3.1 Preliminary data screening and CMB test

The collected data was cleaned and screened. As all data were collected via a single instrument, we tested for Common Method Bias (CMB). A preliminary Harman’s single-factor test was conducted, and to more rigorously assess this, we also employed a CFA-based marker variable technique (Jakobsen and Jensen, 2015), both of which confirmed that method variance was not a significant threat in this study.

#### 3.3.2 Test for hierarchical data effects

Given that the data were collected from 15 different universities, we examined the potential for a data nesting effect. We calculated the Intra-class Correlation Coefficient (ICC(1)) for the key dependent variable, Behavioral Intention (BI). The resulting ICC(1) value was 0.021, which is well below the threshold where multilevel modeling is typically recommended (Mehta et al., 2018), justifying the use of a traditional single-level SEM.

#### 3.3.3 Measurement model analysis

A Confirmatory Factor Analysis (CFA) was conducted on the full measurement model. We deliberately chose this holistic approach, specifying all 11 latent constructs to covary freely, as it provides the most stringent test of the model’s overall structure and discriminant validity (Brown and Moore, 2012). We first report the overall measurement model fit indices (e.g., χ^2^/df, CFI, TLI, RMSEA). We then present the standardized factor loadings, Composite Reliability (CR), and Average Variance Extracted (AVE) to assess internal consistency and convergent validity. Discriminant validity will be assessed by comparing the square root of each construct’s AVE with its correlations with all other constructs.

#### 3.3.4 Structural model and multi-group analysis

After establishing the measurement model’s validity, the structural model was tested to validate the 17 proposed hypotheses. Furthermore, a multi-group SEM analysis was conducted to examine the moderating effects. Crucially, prior to testing the structural paths across groups, we established measurement invariance (configural, metric, and scalar) to ensure that the constructs were measured equivalently, a prerequisite for meaningful group comparisons (Schmitt and Kuljanin, 2008).

### 3.4 Ethical considerations

This research was conducted in strict adherence to all academic ethical standards and received formal approval from Xi’an Jiaotong University. We implemented several procedural and ethical safeguards, not only to protect participants’ rights but also to enhance the methodological rigor of our data by actively mitigating potential response biases.

First, to minimize social desirability bias, particularly concerning sensitive constructs like Perceived Ethical Risks, we took several crucial steps. At the outset of the survey, we explicitly guaranteed absolute anonymity and confidentiality, assuring participants that their responses were untraceable and would be used solely for aggregated academic research. We also clearly stated that there were no “right” or “wrong” answers, encouraging them to provide their most candid personal opinions.

Second, given the subject matter of AIGC, we took the extra step of explicitly reassuring participants that their responses would not be used to monitor or evaluate their personal academic behavior, thereby fostering a climate of trust and encouraging truthful self-reporting.

Finally, all participants were fully informed of the research purpose and their right to withdraw at any time without penalty, providing voluntary online informed consent before beginning the survey. All data were stored on an encrypted server in strict compliance with data protection regulations.

## 4 Results

This section details the empirical findings of the study, organized to systematically test the proposed theoretical model. The analytical procedure unfolds in five sequential stages: (1) a series of preliminary diagnostic tests to ensure data integrity; (2) presentation of descriptive statistics and the correlation matrix; (3) a rigorous assessment of the measurement model’s psychometric properties via CFA; (4) the estimation and evaluation of the structural model to test the research hypotheses; and finally, (5) a multi-group analysis to explore the moderating influence of key demographic characteristics.

### 4.1 Preliminary data diagnostics

Prior to hypothesis testing, we conducted three crucial diagnostic checks. First, Harman’s single-factor test was used to assess Common Method Bias. An unrotated EFA on all items showed the first factor explained only 31.7% of the variance, well below the 40% threshold, indicating CMB was not a significant concern. Second, we assessed Multicollinearity by calculating the Variance Inflation Factor (VIF). All VIF values ranged from 1.24 to 2.81, substantially lower than the critical value of 5, confirming the absence of multicollinearity issues. Finally, given data collection from 15 universities, we tested for Hierarchical Data Effects. The ICC(1) for our primary outcome, Willingness to Use, was 0.021, well below the 0.059 threshold, justifying the use of a standard single-level SEM.

### 4.2 Descriptive statistics and correlation analysis

As detailed in Table 3, the descriptive statistics for the 462 participants revealed key insights into their perceptions. The mean score for Social Influence was 3.68, indicating moderate perceived importance. Hedonic Motivation scored 3.75, reflecting that enjoyment is a significant factor. The mean for Anthropomorphism was 3.82, suggesting an appreciation for human-like characteristics. Notably, students expressed relatively high expectations for Generation Quality (mean = 4.05) and lower concern about Ethical Risk Perception (mean = 3.45).

**Table 3:** Descriptive statistics.

Variable	*N*	Min	Max	M	SD	SI	HM	ANT	ERP	AIX	GQ	SA	PE	EE	EMO	WOU
SI	462	1	5	3.68	0.65	1										
HM	462	1	5	3.75	0.70	0.40**	1									
ANT	462	1	5	3.82	0.60	0.41**	0.25*	1								
ERP	462	1	5	3.45	−0.72	−0.25**	−0.12	−0.21*	1							
AIX	462	1	5	3.90	0.58	0.38**	0.35**	0.36**	−0.32**	1						
GQ	462	1	5	4.05	0.62	0.50**	0.45**	0.40**	−0.35**	−0.15	1					
SA	462	1	5	3.60	0.67	0.30**	0.41**	0.30**	−0.27**	−0.12	0.35**	1				
PE	462	1	5	3.80	0.65	0.35**	0.40**	0.42**	−0.38**	0.34**	0.20*	0.38**	1			
EE	462	1	5	3.76	−0.70	−0.42**	−0.38**	−0.40**	0.35**	−0.30**	−0.18*	−0.29**	−0.31**	1		
EMO	462	1	5	3.55	0.74	0.38**	0.40**	0.29**	−0.33**	−0.14	0.30**	0.39**	0.39**	−0.35**	1	
WOU	462	1	5	4.10	0.61	0.45**	0.41**	0.45**	−0.40**	0.25**	0.25**	0.50**	0.50**	−0.42**	0.38**	1

SI, Social Influence; HM, Hedonic Motivation; ANT, Anthropomorphism; ERP, Perceived Ethical Risk; AIX, Algorithmic Explainability; GQ, Generation Quality; SA, Contextual Awareness; PE, Performance Expectancy; EE, Effort Expectancy; EMO, Emotions; WOU, Willingness to Use; *p < 0.05, **p < 0.01.

The correlation analysis provided preliminary support for our hypotheses. Willingness to Use showed strong positive relationships with Social Influence (r = 0.45), Hedonic Motivation (r = 0.41), and Anthropomorphism (r = 0.45). This suggests that peer support and enjoyment enhance adoption intentions. Furthermore, Ethical Risk Perception negatively impacted both Performance Expectancy (r = −0.38) and Willingness to Use (r = −0.40), indicating that ethical concerns suppress acceptance.

### 4.3 Reliability and validity

A CFA was conducted on the full measurement model including all 11 latent constructs simultaneously to ensure a rigorous test. The model demonstrated an excellent fit to the data (χ^2^/df = 2.48, CFI = 0.921, TLI = 0.910, RMSEA = 0.052).

The psychometric properties of the scales were strong. CR values ranged from 0.80 to 0.87, and Cronbach’s *α* coefficients ranged from 0.77 to 0.86, all exceeding the 0.70 threshold and confirming high internal consistency. For validity, all standardized factor loadings were significant and ranged from 0.75 to 0.88. The AVE for each construct ranged from 0.56 to 0.68, surpassing the 0.50 benchmark. Finally, discriminant validity was established as the square root of each construct’s AVE was greater than its correlation with any other construct. These results, detailed in Table 4, confirm the measures are reliable and valid.

**Table 4:** Reliability and validity.

Variable	Factor loadings	CR	AVE	Cronbach’s α
Social influence	0.75, 0.80, 0.85	0.84	0.61	0.80
Hedonic motivation	0.78, 0.82, 0.79	0.83	0.63	0.77
Anthropomorphism	0.76, 0.81, 0.83	0.85	0.63	0.86
Ethical risk perception	0.77, 0.83, 0.88	0.86	0.65	0.82
Algorithmic interpretability	0.73, 0.76, 0.79	0.80	0.56	0.78
Generation quality	0.79, 0.84, 0.85	0.87	0.68	0.84
Context-awareness	0.80, 0.82, 0.86	0.80	0.68	0.83
Performance expectancy	0.78, 0.79, 0.85	0.84	0.65	0.79
Effort expectancy	0.77, 0.80, 0.84	0.84	0.64	0.85
Emotions	0.75, 0.78, 0.80	0.82	0.60	0.80
Willingness to use	0.76, 0.79, 0.81	0.82	0.61	0.81

### 4.4 Structural model and hypothesis testing

The structural model also showed an excellent fit (χ^2^/df = 2.10, CFI = 0.94, TLI = 0.93, RMSEA = 0.04), with substantial explanatory power for Performance Expectancy (R^2^ = 58%), Effort Expectancy (R^2^ = 51%), and Willingness to Use (R^2^ = 62%).

In predicting Performance Expectancy, Generation Quality had the strongest positive effect (*β* = 0.53, *p* < 0.001), followed by Algorithmic Interpretability (*β* = 0.41, *p* < 0.001) and Context-awareness (*β* = 0.39, *p* < 0.01). Hedonic Motivation (*β* = 0.27, *p* < 0.001) and Anthropomorphism (*β* = 0.21, *p* = 0.01) also had significant positive effects. Conversely, Ethical Risk Perception had a significant negative effect (*β* = −0.25, *p* < 0.01). The path from Social Influence was not significant (*β* = 0.11, *p* = 0.08).

In predicting Effort Expectancy, Generation Quality (*β* = −0.42, *p* < 0.001), Algorithmic Interpretability (*β* = −0.39, *p* < 0.001), Context-awareness (*β* = −0.35, *p* < 0.01), and Anthropomorphism (*β* = −0.33, *p* < 0.01) all had significant negative effects, indicating they reduce perceived difficulty. Ethical Risk Perception significantly increased perceived difficulty (*β* = 0.33, *p* < 0.01). The paths from Hedonic Motivation (*β* = −0.12, *p* = 0.18) and Social Influence (*β* = 0.10, *p* = 0.20) were not significant.

**Figure 2:**
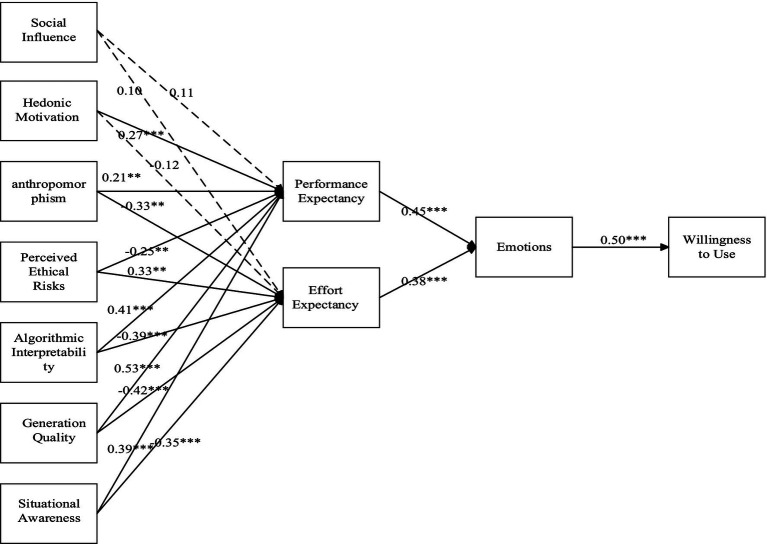
Path coefficient diagram. *p < 0.01, ***p < 0.001.

Finally, Performance Expectancy (*β* = 0.45, *p* < 0.001) and Effort Expectancy (*β* = 0.38, *p* < 0.001) both positively influenced Emotions, which in turn had a strong positive effect on Willingness to Use (*β* = 0.50, *p* < 0.001) (see Figure 2).

### 4.5 Multi-group analysis

Before testing the structural model across groups, we rigorously assessed the measurement invariance of our constructs. As detailed in Table 5, we followed a multi-step process, evaluating configural, metric, and scalar invariance for each demographic variable. The results provide strong support for measurement invariance: for all comparisons, the change in the Comparative Fit Index (ΔCFI) was well below the established threshold of 0.010, and the change in the Root Mean Square Error of Approximation (ΔRMSEA) was below 0.015 (Cheung and Rensvold, 2002). This robustly establishes that the measurement model operates equivalently across the compared groups. Having confirmed this crucial prerequisite, we proceeded with the multigroup path analysis to formally test for significant differences in the key structural relationships.

**Table 5:** Non-variant test.

Grouping variable	Invariance level	χ^2^	df	CFI	TLI	RMSEA	Model comparison	Δχ^2^	Δdf	ΔCFI	ΔRMSEA
Gender (male/female)	Model 1: Configural	1185.3	478	0.928	0.915	0.041					
	Model 2: Metric	1201.7	499	0.923	0.912	0.043	vs. Model 1	16.4*	21	−0.005	+0.002
	Model 3: Scalar	1225.1	520	0.919	0.910	0.044	vs. Model 2	23.4*	21	−0.004	+0.001
Academic discipline (HSS/E&S)	Model 1: Configural	1179.9	478	0.926	0.913	0.040					
	Model 2: Metric	1195.2	499	0.922	0.911	0.042	vs. Model 1	15.3*	21	−0.004	+0.002
	Model 3: Scalar	1219.8	520	0.918	0.909	0.043	vs. Model 2	24.6*	21	−0.004	+0.001
Ethnicity (Han/minority)	Model 1: Configural	1192.4	478	0.930	0.918	0.042					
	Model 2: Metric	1205.1	499	0.927	0.916	0.043	vs. Model 1	12.7*	21	−0.003	+0.001
	Model 3: Scalar	1229.3	520	0.923	0.914	0.044	vs. Model 2	24.2*	21	−0.004	+0.001
Political affiliation (party/non-party)	Model 1: Configural	1168.1	478	0.932	0.921	0.039					
	Model 2: Metric	1188.5	499	0.926	0.916	0.041	vs. Model 1	20.4*	21	−0.006	+0.002
	Model 3: Scalar	1215.9	520	0.921	0.913	0.043	vs. Model 2	27.4*	21	−0.005	+0.002

HSS, Humanities & Social Sciences; E&S, Engineering & Science. Model 1 tests configural invariance (baseline model). Model 2 tests metric invariance by constraining factor loadings. Model 3 tests scalar invariance by constraining item intercepts. Invariance is supported when ΔCFI ≤ 0.010 and ΔRMSEA ≤ 0.015. *The χ^2^ difference (Δχ^2^) was not significant at *p* < 0.05, providing additional support for invariance.

Below, we report the detailed path coefficients for each subgroup analysis, as presented in Table 6, highlighting only the statistically significant differences between groups for clarity.

**Table 6:** Multigroup analysis.

Path	Male	Female	*z*-test (M vs F)	HSS	E&S	*z*-test (HSS vs E&S)	Han	Minority	*z*-test (Han vs Min)	Party	Non-Party	*z*-test (P vs NP)
H1: SI → PE	0.32*	0.46***	−2.11*	0.35**	0.42***	−1.19	0.50***	0.38*	2.31*	0.48***	0.35**	1.85
H2: SI → EE	0.28	0.30*	−0.25	0.25	0.22	0.31	0.32**	0.20	1.76	0.36**	0.24*	1.48
H3: HM → PE	0.44***	0.36**	1.34	0.50***	0.39**	2.25*	0.47***	0.35**	1.88	0.52***	0.31***	3.12**
H4: HM → EE	0.22	0.35**	−1.88	0.31**	0.29	0.22	0.30*	0.20	1.22	0.27*	0.23*	0.49
H5: ANT → PE	0.50***	0.40***	1.58	0.45***	0.38**	1.15	0.42**	0.30*	1.66	0.48***	0.35**	1.78
H6: ANT → EE	0.30*	0.25	0.67	0.27	0.20	1.01	0.28*	0.15	1.89	0.30*	0.18	1.45
H7: ERP → PE	−0.40***	−0.32**	−2.04*	−0.35***	−0.28*	−0.98	−0.38**	−0.22	−2.58**	−0.30**	−0.25*	−0.76
H8: ERP → EE	0.25	0.30**	−0.61	0.20	0.15	0.59	−0.28**	−0.15	−1.84	−0.21*	−0.19	−0.33
H9: AIX → PE	0.45***	0.38**	1.22	0.42***	0.50***	−1.40	0.35**	0.24*	1.77	0.40***	0.28**	1.90
H10: AIX → EE	−0.20	−0.30**	1.49	−0.25*	−0.28**	0.43	0.40***	0.33**	1.41	0.45***	0.38***	1.59
H11: GQ → PE	0.50***	0.46**	0.70	0.52***	0.49***	0.54	0.48***	0.35**	1.94	0.55***	0.39***	2.67**
H12: GQ → EE	−0.35**	−0.25*	−1.35	−0.30*	−0.22	−1.10	0.31**	0.20	1.80	0.38***	0.25**	1.85
H13: SA → PE	0.42***	0.39**	0.51	0.48***	0.37*	1.83	0.20*	0.15	0.99	0.25**	0.18*	1.25
H14: SA → EE	−0.30*	−0.20	−1.29	−0.28	−0.25*	−0.38	0.24*	0.10	1.91	0.28**	0.15	1.88
H15: PE → EMO	0.48***	0.42***	1.18	0.50***	0.45***	0.98	0.56***	0.50***	1.52	0.62***	0.50***	1.78
H16: EE → EMO	0.25*	0.30**	−0.71	0.28**	0.22	1.07	0.41***	0.33**	1.63	0.45***	0.30**	1.95
H17: EMO → WOU	0.50***	0.45***	0.98	0.48***	0.40***	1.57	0.74***	0.68**	1.55	0.79***	0.65***	1.81

SI, Social Influence; HM, Hedonic Motivation; ANT, Anthropomorphism; ERP, Perceived Ethical Risk; AIX, Algorithmic Explainability; GQ, Generation Quality; SA, Contextual Awareness; PE, Performance Expectancy; EE, Effort Expectancy; EMO, Emotions; WOU, Willingness to Use. The table reports standardized path coefficients (*β*) and their significance. The *z*-test column shows the critical ratio for the pairwise path comparison. Significance levels: * *p* < 0.05, ** *p* < 0.01, *** *p* < 0.001. Significant differences in *z*-tests (*p* < 0.05) are highlighted in bold.

#### 4.5.1 Gender differences

The moderating effect of gender was found to be significant on specific paths. The path from Social Influence to Performance Expectancy was significantly stronger for females (*β* = 0.46, *p* < 0.001) than for males (*β* = 0.32, *p* < 0.05), as confirmed by a significant cross-group difference test (*z* = −2.11, *p* < 0.05). Conversely, the negative impact of Perceived Ethical Risk on Performance Expectancy was significantly stronger for males (*β* = −0.40, *p* < 0.001) than for females (*β* = −0.32, *p* < 0.01), a difference that was also statistically significant (*z* = −2.04, *p* < 0.05). While the effect of Hedonic Motivation on Performance Expectancy appeared more pronounced for males (*β* = 0.44, *p* < 0.001) compared to females (*β* = 0.36, *p* < 0.01), the direct comparison of the paths did not reveal a statistically significant difference (*z* = 1.34, *p* > 0.05).

#### 4.5.2 Academic discipline differences

The analysis revealed significant moderation by academic discipline. The effect of Hedonic Motivation on Performance Expectancy was significantly stronger for students in Humanities & Social Sciences (*β* = 0.50, *p* < 0.001) compared to those in Engineering & Science (*β* = 0.39, *p* < 0.01), as supported by the significant path difference (*z* = 2.25, *p* < 0.05). In contrast, while the effect of Social Influence on Performance Expectancy appeared stronger for Engineering & Science students (*β* = 0.42, *p* < 0.001) than for Humanities & Social Sciences students (*β* = 0.35, *p* < 0.01), this difference was not statistically significant (*z* = −1.19, *p* > 0.05).

#### 4.5.3 Ethnic background differences

Ethnicity also emerged as a significant moderator. The effect of Social Influence on Performance Expectancy was significantly higher for Han students (*β* = 0.50, *p* < 0.001) than for Minority students (*β* = 0.38, *p* < 0.05), with the group difference being statistically significant (*z* = 2.31, *p* < 0.05). Furthermore, the negative impact of Perceived Ethical Risk on Performance Expectancy was significant for Han students (*β* = −0.38, *p* < 0.01) but not for Minority students (*β* = −0.22, *p* > 0.05), and this cross-group difference was statistically significant (*z* = −2.58, *p* < 0.01).

#### 4.5.4 Party membership differences

Party membership demonstrated a consistent moderating influence. For instance, the effect of Hedonic Motivation on Performance Expectancy was significantly stronger for Party members (*β* = 0.52, *p* < 0.001) than for non-Party members (*β* = 0.31, *p* < 0.001), a difference confirmed to be significant (*z* = 3.12, *p* < 0.01). Likewise, the impact of Generation Quality on Performance Expectancy was significantly more pronounced for Party members (*β* = 0.55, *p* < 0.001) compared to non-Party members (*β* = 0.39, *p* < 0.001), with the difference being statistically significant (*z* = 2.67, *p* < 0.01).

## 5 Discussion

This study aimed to investigate the complex factors influencing students’ acceptance of AIGC. To this end, we constructed an integrative theoretical model that uses the AIDUA model as its content framework and innovatively employs CAT as its core processing mechanism, thereby opening the “black box” of traditional acceptance models. To ensure the framework accurately captures the unique characteristics of AIGC, we further strategically integrated two key dimensions: a technical performance dimension (Generation Quality; Context-awareness) and a socio-ethical dimension (Perceived Ethical Risk; Algorithmic Explainability). The empirical results not only validate the structural integrity of this integrative model but also yield profound insights into the interplay among technical attributes, individual psychological appraisals, and key user characteristics. This section is organized around our most significant empirical findings to elaborate on their theoretical and practical implications.

### 5.1 Interpretation of key findings

The findings of this study lend strong support to our proposed integrative model and unveil the complex cognitive appraisal mechanisms at play in the AIGC acceptance process.

#### 5.1.1 The overwhelming influence of AIGC-specific dimensions: a dual appraisal of technology and ethics

The most significant finding of this study is that AIGC-specific technical performance and socio-ethical dimensions exhibit a much stronger predictive power on students’ core beliefs than the classic antecedents found in traditional acceptance models (e.g., social influence, hedonic motivation). Specifically, Generation Quality and Context-awareness are the strongest drivers for enhancing performance expectancy, confirming our core hypothesis.

This finding stands in stark contrast to prior research on general-purpose software or systems, where performance expectancy is often driven more by external factors like social influence or organizational mandates (Budhathoki et al., 2024). Our results, however, indicate that for intelligent technologies like AIGC, which are centered on content output, the focus of user evaluation shifts from “external environmental pushes” to the “strength of the technical core.” This confirms and extends the classic assertion that perceived usefulness is paramount, specifying that its core meaning in the AIGC era is the capacity for high-quality generation.

Simultaneously, Perceived Ethical Risk exerted a significant negative influence on both performance and effort expectancy, while Algorithmic Explainability dually enhanced performance expectancy and reduced effort expectancy.

This result substantially enriches existing technology acceptance theories. Traditional models rarely incorporate ethical considerations. Although some scholars have recently called for integrating trust or risk into these models (Slade et al., 2015), they are often treated as single, monolithic variables. By operationalizing the ethical dimension into “perceived risk” and “explainability” and demonstrating their independent, powerful predictive force on core beliefs, our study robustly answers these calls. This suggests that in the age of AI, ethical considerations are no longer secondary factors but have become core antecedents, equal in importance to technical performance, in determining user adoption—a significant revision and supplement to traditional acceptance models.

This shift is so profound that it appears to have overshadowed the influence of some classic antecedents. Notably, our structural model revealed that the direct paths from Social Influence to both Performance Expectancy and Effort Expectancy were non-significant. This finding, a stark deviation from the core tenets of models like UTAUT, does not imply that social cues are irrelevant. Rather, it suggests that when facing a technology whose performance and risks can be directly and immediately experienced, users prioritize their first-hand cognitive appraisal of the tool’s core functionality over vicarious information. In the AIGC context, what the tool can do (Generation Quality) and what risks it might pose (Ethical Risk) become far more salient informational cues than what others say about it. This provides a clear boundary condition for the applicability of traditional acceptance models in the era of powerful, experience-driven AI.

#### 5.1.2 The influence of classic antecedents and cognitive mediation

The pathways for the three classic antecedents—social influence, hedonic motivation, and anthropomorphism—remain clear, perfectly corroborating the explanatory power of CAT. For instance, social influence affects both performance expectancy (H1) and effort expectancy (H2).

This dual-influence pathway confirms findings from prior research, but our study provides a deeper psychological mechanism by introducing CAT. Whereas traditional research merely depicted the “social influence → core beliefs” link, our model reveals that this association is underpinned by users’ simultaneous cognitive processing of “benefit appraisal” (primary appraisal) and “resource appraisal” (secondary appraisal). This opens the “black box” for understanding how social influence is specifically translated into personal beliefs.

#### 5.1.3 The complete pathway: core beliefs, affect, and final acceptance intention

The results fully validated the mediating pathway from core beliefs to affect and, ultimately, to acceptance intention, with affect playing a crucial mediating role.

The findings of this study reaffirm the critical role of affect in technology acceptance, a conclusion highly consistent with prior research which also identified affect as a vital bridge between cognitive appraisal and final behavior (Smith and Kirby, 2012). However, our study validates this finding in the novel and highly interactive context of AIGC, pointing out that performance expectancy (rather than effort expectancy) is the primary source of positive affect. This may imply that for AIGC users, the emotional experience stems more from the surprise and satisfaction of “achieving unexpectedly good results” than merely from the fluency of “effortless operation.” Intriguingly, and in seeming contradiction to traditional usability tenets, our results showed that Effort Expectancy positively influenced Emotion. This counter-intuitive finding suggests that the meaning of “effort” may be reappraised in the context of creative or intellectual partnership with AI. Instead of being a pure “cost” to be minimized, the cognitive effort invested in mastering prompt engineering or co-creating with AIGC could be perceived as a form of rewarding engagement. The process of overcoming a moderate level of difficulty to achieve a desired output can foster a sense of competence and accomplishment, thereby generating positive affect. This implies that for advanced AI tools, the goal may not be to eliminate effort entirely, but to design an optimally challenging and intellectually stimulating user experience.

#### 5.1.4 The moderating role of individual differences: a contextualized understanding of AIGC acceptance

The results of the multi-group analysis revealed heterogeneity within the student population, confirming the significant moderating role of individual differences in the AIGC cognitive appraisal process, while also yielding some unexpected findings.

For instance, while our analysis confirmed gender differences, it unveiled a nuanced picture that challenges common assumptions. The negative impact of Perceived Ethical Risk on Performance Expectancy was significantly stronger for males than for females. This counter-intuitive result may suggest that male students, perhaps adopting a more instrumental view, are quicker to downgrade their assessment of a tool’s utility once they perceive its ethical flaws (e.g., potential for plagiarism, inaccurate outputs) as a direct threat to achieving a reliable outcome. In contrast, the finding that females were more strongly influenced by Social Influence aligns with established literature, but our study situates this within the AIGC context, highlighting the persistent role of social networks in shaping female students’ initial technology appraisals.

Beyond these general demographic factors, the influence of individual differences becomes even more pronounced when examining variables unique to the Chinese context. While the influence of individual differences in technology acceptance is well-supported by a large body of literature (Xiao and Sun, 2022; Kosiara-Pedersen et al., 2025), this study provides unique, context-rich insights by employing academic background and political affiliation as moderators. The sensitivity differences between STEM and humanities students regarding technical and ethical dimensions, in particular, not only confirm prior theories on cognitive style differences (Tsang, 2019) but, more importantly, propose a novel, fine-grained perspective for promoting AIGC in education: a one-size-fits-all promotion strategy is ineffective. Instead, guidance must be tailored to the “cognitive paradigms” of different disciplines.

An equally noteworthy finding is the absence of statistically significant differences between Han and ethnic minority students, as well as among students of different grade levels. This “null result” is itself highly instructive. It may suggest that within China’s current highly integrated and information-centric educational environment, the influence of AIGC as a new, pervasive learning tool transcends traditional ethnic-cultural backgrounds and simple grade-level distinctions. For contemporary university students, who share similar digital life environments and academic pressures, this common identity as “digital natives” may have a stronger influence than their ethnic or grade-level affiliations when confronting a general-purpose technology like AIGC. This implies that researchers and practitioners, when considering AIGC adoption, should focus more on the “cognitive paradigms” shaped by academic disciplines rather than over-relying on traditional demographic classifications.

### 5.2 Theoretical implications

This study contributes several key theoretical insights to the fields of technology acceptance and human-computer interaction:

Proposing an Integrative AI Acceptance Model: The primary contribution of this research is the successful integration of the content variables of the AIDUA model with the processing mechanisms of CAT, extended to address the unique characteristics of AIGC. This layered, integrative model explains not only “what” influences acceptance intention but, more critically, “how” it influences, providing a theoretical framework with greater explanatory power for understanding user acceptance of complex AI technologies.
Empiricizing and Integrating the Ethical Dimension into an Acceptance Model: Past TAMs have largely focused on the utilitarian and ease-of-use aspects of a tool. This study is the first to incorporate “perceived ethical risk” and “algorithmic explainability” as core variables and to demonstrate with empirical data their strong predictive power on user’s core beliefs. This moves the paradigm of technology acceptance theory from a “human-computer” dyadic interaction toward a “human-computer-society” triadic cognitive framework.
Deepening the Application of CAT: This research transforms CAT from a general psychological theory into an analytical tool capable of explaining specific pathways in technology acceptance. By conceptualizing antecedents as “cues to be appraised” and core beliefs as the “outcomes of appraisal,” we offer a robust theoretical pathway for future research on how to introduce new contextual variables.

### 5.3 Practical implications

The findings of this study offer significant practical guidance for AIGC designers, educational policymakers, and front-line educators:

For AIGC developers: Technology and ethics must be twin-driven. While iterating algorithms to improve generation quality and context-awareness, developers must place equal strategic importance on enhancing algorithmic explainability and reducing users’ perception of ethical risk. Features such as “one-click source tracing,” “citation suggestions,” and “risk alerts” may no longer be nice-to-haves but are essential elements for winning user trust and improving product competitiveness.
For educational policymakers and administrators: Clear AIGC usage norms and guidelines should be established. Given students’ high sensitivity to ethical risks, schools and educational authorities should promptly issue guidelines on the use of AIGC in academic activities, clarifying boundaries to mitigate the academic integrity risks students perceive due to uncertainty.
For front-line teachers: Adopt differentiated, guided teaching strategies. Teachers should recognize the cognitive differences among students from various academic backgrounds. For STEM students, the focus could be on guiding them to reflect on the ethical and social impacts behind the technology. For humanities and social science students, the emphasis could be more on demonstrating how to leverage AIGC as a tool to enhance academic productivity. Offering specialized seminars or workshops to improve students’ “AI literacy” is key to bridging cognitive divides and fostering the healthy development of AIGC in education.

## 6 Limitations and future research directions

While this study offers a robust and nuanced model of AIGC adoption, its conclusions must be framed by its inherent limitations. These limitations, however, are not mere methodological footnotes; they are generative, pointing directly toward a more ambitious and sophisticated future research agenda.

### 6.1 Limitations rooted in our findings

#### 6.1.1 Cultural and contextual specificity

A primary and acknowledged limitation is that our sample, while diverse across 15 institutions, was drawn exclusively from China. This necessarily bounds the cross-cultural generalizability of our findings. Key cultural dimensions, such as collectivism, power distance, and specific educational norms prevalent in China, may significantly shape how students perceive factors like social influence and ethical risk. For instance, the non-significant path from Social Influence to Effort Expectancy might yield different results in a more individualistic cultural context. Therefore, while our model provides a robust theoretical baseline for the cognitive appraisal process, its specific path coefficients demand cautious interpretation and invite future cross-cultural validation to test its applicability in Western and other non-Chinese educational systems. Acknowledging this boundary condition, we now turn to the limitations inherent in the model’s theoretical and methodological design.

#### 6.1.2 The “cognitive appraisal” black box

Our model, grounded in CAT, successfully links technological affordances to cognitive evaluations (e.g., Generation Quality → Performance Expectancy). However, our quantitative design treats the appraisal process itself as a “black box.” We do not capture the live, dynamic, and often messy thought processes students engage in when they weigh, for instance, the instrumental benefits of a high-quality output against the ethical unease it provokes. Qualitative methods, such as think-aloud protocols or digital ethnography, are needed to pry open this black box and observe the appraisal process *in situ*.

#### 6.1.3 The assumption of a stable “ethical risk” construct

We operationalized Ethical Risk Perception as a single, static construct. This overlooks its potential multi-dimensionality. Is the “risk” perceived by students primarily about academic integrity (plagiarism), data privacy, or the veracity of AI-generated information (misinformation)? These distinct facets of risk may trigger different appraisal pathways and coping responses. Our model’s parsimony in this regard may mask deeper, more specific anxieties that warrant their own lines of inquiry.

#### 6.1.4 The intention-behavior gap and social desirability bias

In line with established models, we used Willingness to Use as a proxy for actual behavior. This is a well-accepted methodological choice, but it carries a notable limitation amplified by the controversial nature of AIGC. The gap between intending to use AIGC responsibly and the actual practice of doing so is likely significant. This is compounded by a potential social desirability bias, where participants may report ethically-aligned intentions because they perceive them as the “correct” or socially approved answers. Consequently, our reliance on self-reported intentions might present an overly optimistic view of student behavior. The real-world challenges—such as corner-cutting under pressure, over-reliance on imperfect outputs, or uncritical acceptance of information—may not be fully captured. Our cross-sectional design cannot bridge this crucial intention-behavior gap, which is central to the ultimate educational impact of AIGC and represents a key avenue for future observational or behavioral research.

### 6.2 A vision for the next generation of AIGC research

The limitations of our study and the dynamic nature of AI in education illuminate several urgent avenues for future inquiry. These move beyond simple model extensions toward a more robust and critical research agenda.

First, future research must go from cross-sectional snapshots to longitudinal “adoption journeys.” A student’s relationship with AIGC is not a static event but an evolving process. To truly understand this, we need longitudinal studies that track how students’ perceptions of Ethical Risk and Generation Quality change after a semester of sustained use. This approach allows us to map the “adoption trajectories” that reveal the dynamic interplay between users and technology over time.

Second, and in direct response to the need for causal evidence, future work must incorporate rigorous experimental designs. Our correlational model has identified what matters; experiments can tell us how to intervene effectively. Building on our findings, two specific experimental paths are particularly promising:

#### 6.2.1 Intervention studies on “ethical literacy”

Researchers should conduct controlled experiments to test the causal impact of the educational programs we recommend. By measuring pre- and post-intervention changes in Ethical Risk Perception and Effort Expectancy between a treatment group (receiving ethical training) and a control group, we can empirically validate the most effective pedagogical strategies.

#### 6.2.2 Controlled experiments on “explainable AI” (XAI)

To test the importance of Algorithmic Interpretability, studies could present participants with different AIGC interfaces, systematically varying the level of explainability (e.g., no source vs. source-linking). This would allow for precise measurement of how XAI features causally affect user trust and Performance Expectancy.

Finally, the ultimate goal of our field should be to move from studying “acceptance” to understanding “critical appropriation.” The key question is not if students use AIGC, but how wisely they integrate it into their intellectual workflows. This calls for a paradigm shift toward developing and validating new constructs that measure concepts like “Reflective AIGC Use” or “Strategic Prompting.” Such work is essential for guiding education toward a future where AI is not just accepted, but critically and productively appropriated.

## Data Availability

The original contributions presented in the study are included in the article/supplementary material, further inquiries can be directed to the corresponding author.
